# Intestinal colonization with ESBL-producing *Klebsiella pneumoniae* in healthy rural villager: A genomic surveillance study in China, 2015-2017

**DOI:** 10.3389/fpubh.2022.1017050

**Published:** 2022-12-15

**Authors:** Shuang Wang, Hengjie Xie, Yuzhen Chen, Lu Liu, Ming Fang, Dapeng Sun, Liuchen Xu, Zhenqiang Bi, Gaoxiang Sun, Yan Li, Xiaolin Yu, Huaning Zhang, Zengqiang Kou, Beiwen Zheng

**Affiliations:** ^1^Infection Disease Control of Institute, Shandong Center for Disease Control and Prevention, Jinan, Shandong, China; ^2^Department of Supervise Sampling, Shandong Institute for Food and Drug Control, Jinan, Shandong, China; ^3^State Key Laboratory for Diagnosis and Treatment of Infectious Disease, Collaborative Innovation Center for Diagnosis and Treatment of Infectious Diseases, The First Affiliated Hospital, College of Medicine, Zhejiang University, Hangzhou, Zhejiang, China; ^4^Jinan Microecological Biomedicine Shandong Laboratory, Department of Structure and Morphology, Jinan, Shandong, China; ^5^Research Units of Infectious Diseases and Microecology, Chinese Academy of Medical Sciences, Beijing, China

**Keywords:** intestinal colonization, ST101, CTX-M-3, core-genome single nucleotide polymorphisms, ESBL-producing *K. pneumoniae*

## Abstract

**Background:**

The worldwide emergence and diffusion of extended-spectrum β-lactamase-*K. pneumoniae* (ESBL-KP) is of particular concern. Although ESBL-KP can inhabit the human gut asymptomatically, colonization with ESBL-KP is associated with an increased risk of ESBL-KP infection and mortality. In this study, we investigated the prevalence and characteristics of ESBL-KP in fecal samples from healthy persons in 12 villages in Shandong Province, China.

**Methods:**

Screening for ESBL-KP in fecal samples was performed by selective cultivation. The bacterial species were identified using matrix-assisted laser desorption/ionization time-of-flight mass spectrometry (MALDI-TOF MS) and 16S rDNA sequence analysis. Minimum inhibitory concentrations (MICs) of 16 antibiotics were determined by the agar dilution method. Plasmid replicons, antimicrobial resistance genes and Sequence types (STs) of the isolates were determined by whole-genome sequencing (WGS). Genetic relatedness of ESBL-KP isolates was determined by the single nucleotide polymorphisms (SNP). The S1 nuclease-pulsed-field gel electrophoresis (S1-PFGE) was used to characterize the plasmids carried by ESBL-KP isolates. Conjugation assays was used to verify the transferability of *bla*_CTX − M_.

**Results:**

ESBL-KP prevalence rates increased from 12.0% in 2015 to 27.5% in 2017. The experimental results showed that 97% of isolates had multi-drug resistance. Multiple ESBL resistance genotypes were commonly detected in the isolates. STs among the ESBL-KP isolates were diverse. All 69 *bla*_CTX−M−3_-positive isolates were located on plasmids, and these genes could be transferred with plasmids between different strains. Phylogenetic analysis showed the possibility of transmission among some isolates.

**Conclusion:**

This study obtained the drug resistance patterns, the drug resistance phenotype and molecular characteristics of fecal-derived ESBL-KP in rural communities in Shandong Province, China. We report a rapid increase in occurrence of ESBL-KP among fecal samples collected from healthy rural residents of Shandong Province from 2015 to 2017. The carriage rate of multidrug-resistant bacteria in healthy residents is increasing. Thus, a need for further monitoring and possible interventions of ESBL-KP in this region is warranted.

## Introduction

ESBL-KP is one of the most common multi-drug resistant groups of Enterobacteriaceae worldwide (1). As an essential nosocomial pathogen, its invasive infection recently resulted in high mortality rates (2, 3). Acquired drug resistance in human commensal bacteria such as *Escherichia coli* and *K. pneumoniae* has become a widespread threat to public health (4). Of recent concern is the increasing prevalence and risk factors of ESBL-KP in community-acquired infections (2). ESBLs-producing is the main reason for *K. pneumoniae* developing drug resistance and spreading rapidly and widely. However, the exact situation of ESBL-KP in healthy residents living an ordinary life in the community is still unclear (5, 6).

The global epidemic of ESBLs mainly includes SHV, TEM, and CTX-M types, and in the last two decades, CTX-M has replaced SHV as the significant type of ESBLs disseminating worldwide (2, 7). More than 190 CTX-M variants have been reported worldwide to date (8). CTX-M-1 was the first reported CTX-M enzyme, and since then, many derivatives of CTX-M have been reported around the world. CTX-M-33, a point mutation derivative of CTX-M-15, was identified in *K. pneumoniae* from a patient hospitalized in Portugal. It is the first CTX-M enzyme possessing a weak carbapenemase activity (9). In Turkey, *E. coli* isolates from chicken meats were found to carry predominantly CTX-M-1 genes, followed by CTX-M-89 and CTX-M-2 (10). Recently, CTX-M-167 positive *K. pneumoniae* was identified for the first time in China (11). While *bla*_CTX−M−15_ and *bla*_CTX−M−14_ are identified as the most prevalent ESBL-encoding genes in the world, *bla*_CTX−M−14_ has been identified as the most common ESBL-encoding gene in China (12). The association of *bla*_CTX−M_ genes with mobile genetic elements, epidemic plasmids, and successful clones ensured rapid and wide dissemination that changed the epidemiology of antibiotic resistance worldwide (13).

Conjugative plasmids are one of the most important mechanisms for the appearance and spread of *bla*_CTX−M_. Additionally, these plasmids often encode multiple resistance determinants, including fluoroquinolones, quinolones, trimethoprim/sulfamethoxazole (SXT) and aminoglycosides (14). The study found three incompatibility (Inc) groups of plasmids, the narrow-host-range IncF and IncI1 plasmids and the broad-host-range IncC plasmid, formerly IncA/C2, are prevalent and associated with ESBL-producing Enterobacteriaceae strains (15). The plasmids carrying virulence genes and antimicrobial resistance may now pose a serious threat to public health because these mobile plasmids accelerate the horizontal transfer of these genes between strains of the same or different species (8, 16). Thus, the identification and characterization of these plasmids may facilitate a better understanding of ESBL-KP transmission mechanism and the development of new measures to control ESBL-KP producers in the community.

Our objectives in this study were to retrospectively investigate the drug resistance phenotype and molecular characteristics of human fecal-derived ESBL-KP in a rural community in Shandong Province, China, and to understand ESBL-KP in a Chinese rural community better. The prevalence of *Klebsiella* and its comparison with domestic and foreign genome-wide data were added to the current database. Our results will outline a larger picture of ESBL-KP within the Chinese community and will provide theoretical support for the government to establish a drug-resistant bacteria prevention and control system in the community.

## Materials and methods

### Bacterial isolation and identification

**Figure 1:**
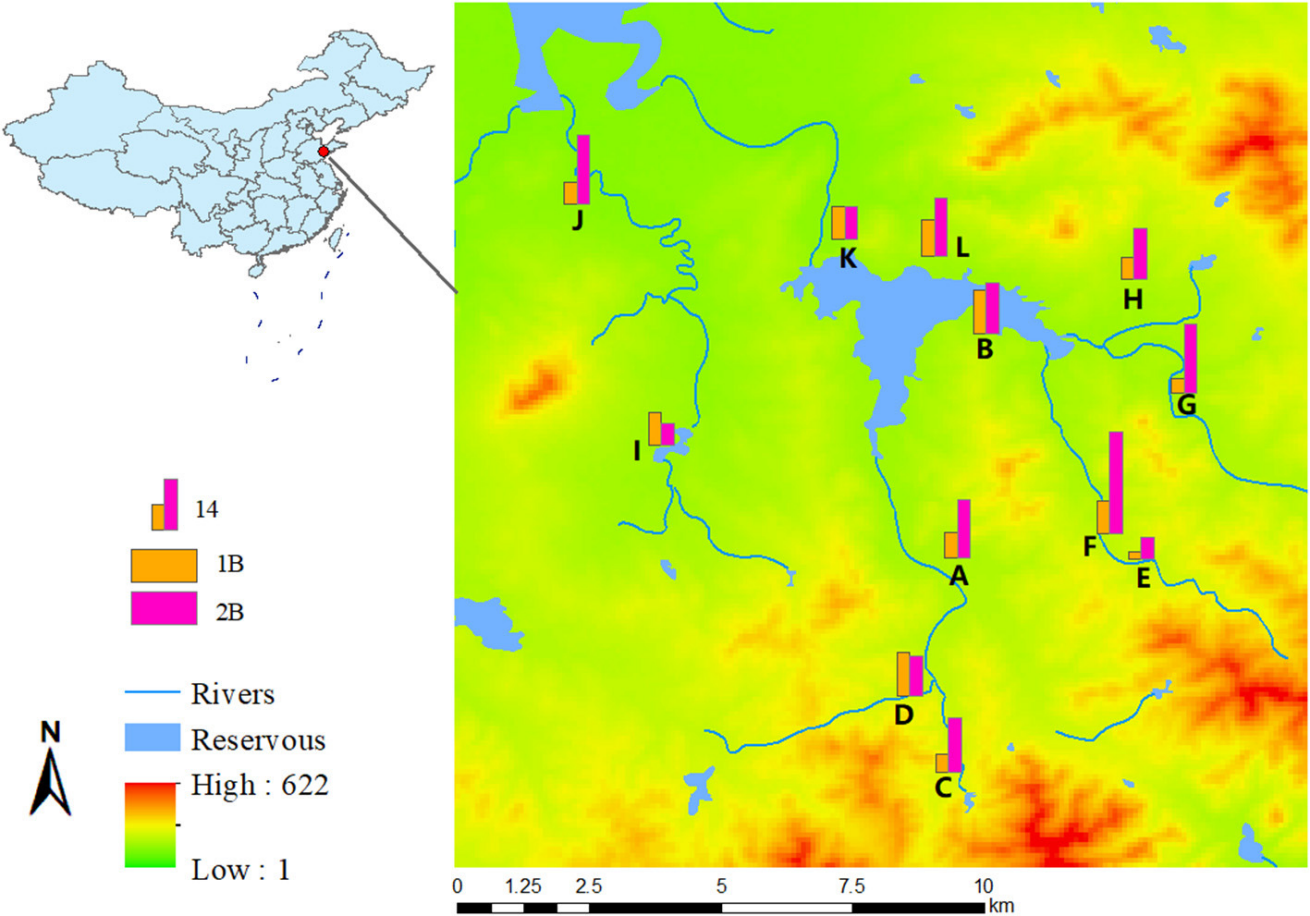
Geographic distribution map of ESBLs-producing *K. pneumoniae* from village A to L, in 2015 and 2017, with digital elevation model.

To better understand the prevalence of ESBL-KP, antimicrobial resistance of bacteria in feces of healthy adults was monitored in Shandong Province, China, in 2015 and 2017 (Figure 1). The selection of villages and participants has been described elsewhere (15). In 2015, 758 households in the study region were selected, and in 2017, 628 of these households were sampled again. One individual from each household was sampled on each occasion. Fecal samples were collected and cultured overnight on chromogenic agar medium (ESBL-Bx, bioMérieux, Marcy l'Etoile, France) at 37°C to screen for ESBL-KP strains. The suspected *K. pneumonia*e colonies identified based on the manual's description were picked and subcultured on the corresponding basal agar at 37°C overnight. Identification of the bacterial species was identified based on 16S rDNA gene sequence analysis and MALDI-TOF MS, as described previously (12). SPSS 22.0 software was used for statistical analysis of the detection rate of each village, and CohranMantel Haenszel was used for stratification Chi-squared test for comparison between groups, *P* < 0.05 indicated that the difference was statistically significant.

### Antimicrobial susceptibility testing

A total of 16 antibiotics were tested. MICs of 14 antimicrobial agents (cefotaxime, ceftazidime, cefoxitin, piperacillin-tazobactam, gentamicin, amikacin, amoxicillin-clavulanate, trimethoprim-sulfamethoxazole, imipenem, meropenem, tetracycline, florfenicol, and furantoin) were determined by agar dilution method, and MICs of two other antimicrobial agents (tigecycline and colistin) were determined by the broth microdilution method. The breakpoints for susceptibility were determined according to Clinical & Laboratory Standards Institute (CLSI, USA) and European Committee on Antimicrobial Susceptibility Testing (EUCAST, Europe). ESBL phenotype was confirmed by the double-disk diffusion method with incorporate cefotaxime and ceftazidime tested alone and combined with clavulanate, according to CLSI. The above experiments used *E. coli* ATCC25922 as quality control (12). Chi-squared test was used for statistical analysis to compare the difference of drug resistance between the 2 years.

### Whole-genome sequencing

All ESBL-KP strains were subjected to WGS. Genomic DNA was extracted using a commercial kit. Libraries were prepared from genomic DNA according to the manufacturer's protocol and sequenced on an Illumina HiSeq (paired end, 2 × 150 bp). *De novo* assembly was generated by using SPAdes 3.11.0 (17). Plasmid replicons, antimicrobial resistance genes and STs were predicted from the genomes using PlasmidFinder 1.3, ResFinder 2.1, and MLST 2.0, respectively (18). The genetic environment surrounding *bla*_CTX−M_ was annotated using RAST3 and Easyfig 2.2.3 (19). In order to determine the phylogenetic relationships among study isolates, and compared them with isolates from diverse host sources from other regions to place them in the international context. For this purpose, we downloaded genome sequences of 99 ESBL-KP isolates from various regions of the world. Using the kSNP3 analysis software, the kmer_length parameter was set to 13 for SNP analysis and phylogenetic tree construction, and evolview beautified and modified the phylogenetic tree (20).

### S1 nuclease-pulsed-field gel electrophoresis (S1-PFGE) and Southern blotting

The CTX-M-3 enzyme has been widely detected; however, its genetic environment has rarely been well investigated as other prevalent ESBLs. S1-PFGE and Southern blotting were performed to locate *bla*_CTX−M−3_ and determine plasmid size in all *bla*_CTX−M−3_-positive *K. pneumoniae*. Whole-cell DNA of all *bla*_CTX−M−3_-positive isolates was extracted and embedded in agarose gel plugs. The DNA in agarose plugs was treated with S1 nuclease (TaKaRa, Beijing, China), and separated by PFGE. Plasmids obtained by PFGE were transferred horizontally to a nylon membrane and hybridization with labeled probes in an HL-2000 HybriLinker Hybridization Oven (UPV, Germany) (12). Molecular mass standard reference strain was *salmonella enterica* serotype Braenderup strain H9812.

### Conjugation assay

Transfer of antibiotic resistance was studied using conjugation for all ESBL-KP isolates. *E. coli* EC600 (rifampicin-resistant) or *E. coli* J53 (azide-resistant) was used as a recipient strain, whereas all ESBL-KP resistant to cefotaxime served as donors. The experimental details were described in our previous study (12).

### Nucleotide sequence accession number

This whole genome and annotation has been deposited in NCBI GenBank under the accession number of PRJNA833746.

## Results

### ESBL-KP isolations and isolation sites

ESBL-KP was isolated from 91 and 173 individuals in 2015 and 2017, respectively. This corresponds to an increase in ESBL-KP occurrence from 12.0% in 2015 to 27.5% in 2017. The mean age of the study participants was 66 with a range between 20–85 years old, and 53% (*n* = 22) were women. The detection rate of ESBL-KP increased in all but three villages. There were statistical differences in the trend of the detection rate among different villages with the year (χ^2^ = 24.682, *P* = 0.010) (Figure 1).

### Antimicrobial resistance profiles of ESBL-KP isolates

**Figure 2:**
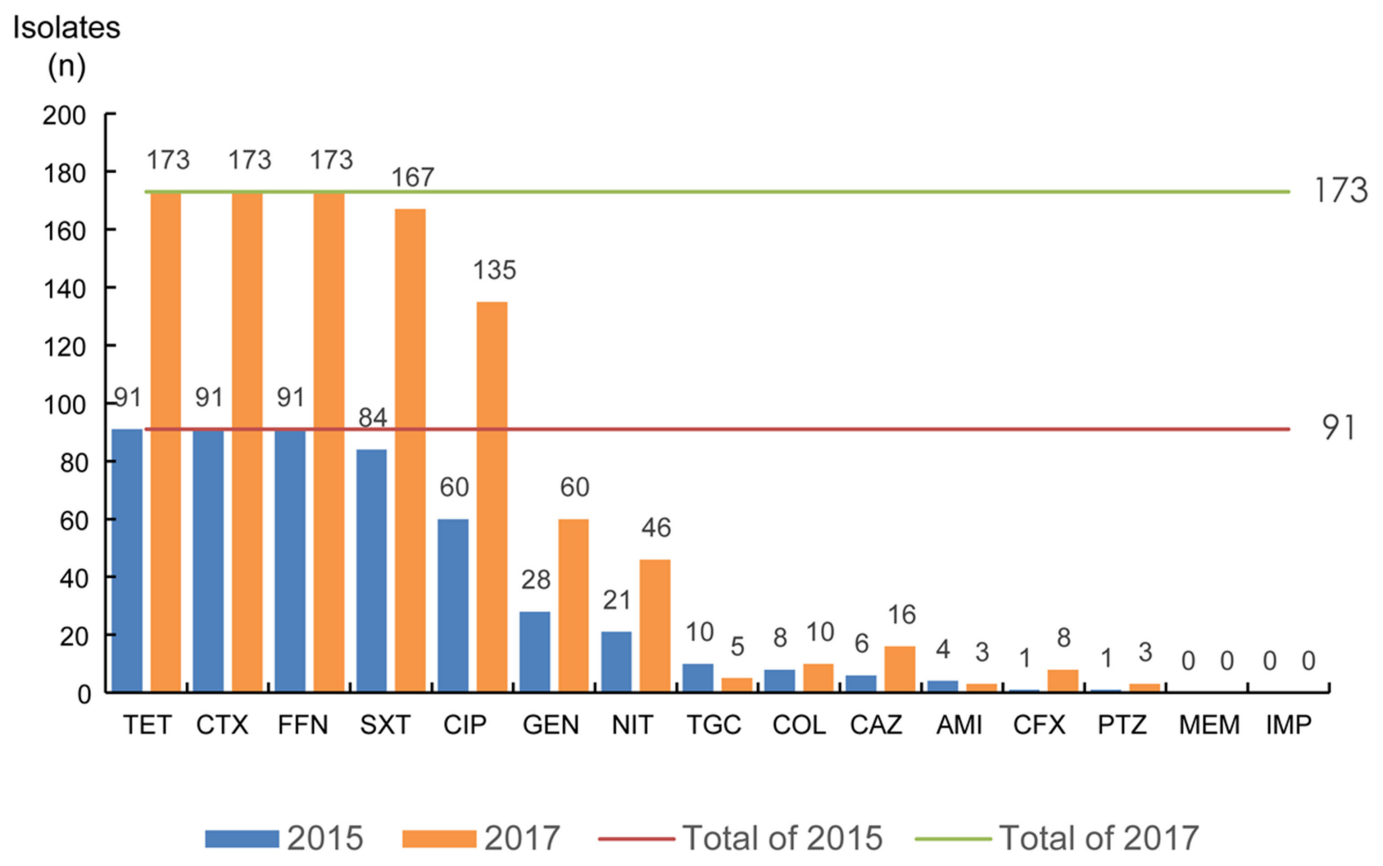
The antimicrobial susceptibility of ESBLs-producing *K. pneumoniae*. TET, tetracycline; CTX, cefotaxime; FFN, florfenicol; SXT, trimethoprim-sulfamethoxazole; CIP, ciprofloxacin; GEN, gentamicin; NIT, furantoin; TGC, tigecycline; COL, colistin; CAZ, ceftazidime; AMI, amikacin; CFX, cefoxitin; PTZ, piperacillin-tazobactam; MEM, meropenem; IMP, imipenem.

All isolates were insensitive to cefotaxime, tetracycline and florfenicol. In addition, susceptibility to ciprofloxacin and trimethoprim-sulfamethoxazole was also lower (< 10%), while susceptibility to meropenem, amikacin, and tigecycline was higher (≥ 90%). Statistically significant differences were observed in susceptibility rates for ciprofloxacin and tetracycline (*p* < 0.05) in 2015 and 2017. The isolates were also generally sensitive to amikacin (95.6%, 98.3%), piperacillin-tazobactam (98.9%, 98.3%), ceftazidime (93.4%, 90.8%), cefoxitin (98.9%, 95.4%) and Colistin (91.2%, 94.02%), however showed lower sensitivity to trimethoprim-sulfamethoxazole (7.7%, 3.5%) (Figure 2). Moreover, the experimental results showed that 97% of isolates have multi-drug resistance (i.e., resistant to antibiotics of more than two classes of antibiotics).

### ESBL-encoding genes

Whole-genome sequencing showed that all ESBL-KP isolates carry ESBL_S_ resistance genes. Various TEM, SHV, and CTX-M types were commonly detected in isolates. The most common ESBL genes were *bla*_TEM−1B_ (124, 47.0%), *bla*_CTX−M−14_ (112, 42.4%), and *bla*_SHV−11_ (86, 32.6%) in all isolates in 2015 and 2017. The coexistence of CTX-M, SHV, and TEM genes was identified in 115 isolates (43.6%). In addition, both CTX-M and SHV co-existed in 103 isolates (39.0%), and both CTX-M and TEM co-existed in 14 isolates (5.3%).

**Figure 3:**
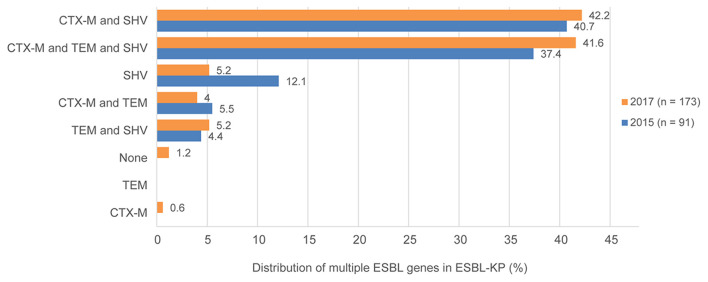
Existence and proportions of multiple ESBL genes for ESBLs-producing *K. pneumoniae* in 2015 and 2017.

The *bla*_CTX−M_ genes were detected in 230 (87.1%) of 264 ESBL-KP isolates. The *bla*_CTX−M−14_ gene was the most prevalent one (*n* = 114), followed by *bla*_CTX−M−3_ (*n* = 70), *bla*_CTX−M−15_ (*n* = 15), *bla*_CTX−M−27_ (*n* = 14), *bla*_CTX−M−24_ (*n* = 7), and *bla*_CTX−M−65_ (*n* = 5), *bla*_CTX−M−55_, and *bla*_CTX−M−5_ (*n* = 2 for each), and *bla*_CTX−M−105_ (*n* = 1). Additionally, both *bla*_CTX−M_ genes co-existed in 5 isolates. The detection of ESBL genes in ESBL-KP in 2015 and 2017 is shown in Figure 3.

### Non-ESBL antimicrobial resistance genes

Plasmid-mediated quinolone resistance (PMQR) genes were identified in all isolates. Apart from *oqxA* and *oqxB*, which were detected in all except four isolates (98.5%), and-type genes (including *qnrB1, qnrB2, qnrB4, qnrB6, qnrB49, qnrB52, qnrB66, qnrS1* and *qnrS2*) were detected in 251 isolates (95.1%), with *qnrS1* being the most abundant and found in 212 isolates (80.3%). Sulphonamide resistance genes were present in 97.7% (*n* = 258) of isolates, with the *sul1* gene as the most prevalent (259, 98.1%). The *sul1* and *sul2* genes often co-occur in the same strain in this study, and the *sul3* gene was detected in three isolates in 2015. Aminoglycoside resistance genes were commonly detected, and 252 isolates (95.5%) carried at least one aminoglycoside resistance gene. The most common were *aac(**3**)-IId* (150, 56.8%), followed by *aac(6')-Ib-cr* (113, 42.8%), *aph(3')-Ib* (68, 25.8%), *aadA16* (107, 40.5%), and *aph(3')-Ia* (69, 26.1%). Notably, the linked strA-strB genes, which confer streptomycin resistance in at least 17 genera of gram-negative bacteria, were detected in 51 isolates (19.3%). A total of 217 isolates were found to carry *tet(A)* (82.2%), followed by *tet(D)* (74, 28.0%) and *tet(G)* (1, 0.38%). All of the isolates carrying *tet*-type genes were resistant to tetracycline. Furthermore, the colistin resistance gene *mcr-1* gene was detected in one isolate recovered in 2015.

### Multilocus sequence determination of the ESBL-KP isolates

The ESBL-KP collected in the study showed high diversity in terms of STs. The 264 isolates displayed 105 different sequence types (ST), and only 9 STs were detected in isolates from both years. ST101 is the most common STs (21, 8.0%), ST37 (16, 6.0%), and ST661 (8, 3.0%) and the three ST isolates together constituted 17.13% of all isolates. These STs were followed in prevalence by ST17, of which seven isolates belonged (2.7%), and ST323, ST896, ST34 and ST1263, for which six isolates of each ST were found (2.2%). Notably, most of the detected STs corresponded to only one isolate representative (*n* = 61).

### Determination of the *bla*_CTX-M-3_ gene location and transferability

In this study, S1-PFGE has confirmed this multi-plasmid nature in at least 35 strains (**Figure 5**). The Southern blot hybridization demonstrated that the *bla*_CTX−M−3_ genes among all 69 *bla*_CTX−M−3_-positive isolates were located on plasmids, the type and size of the plasmid where A is located are shown in Table 1. Interestingly, the *bla*_CTX−M−3_ gene of two isolates was located on two plasmids of different sizes, respectively (Table 1, **Figure 5**). Furthermore, all *bla*_CTX−M−3_ genes could be transferred from the isolate to the recipient by conjugation, and this gene in the recipient was detected by PCR (data not shown).

**Table 1:** Analyses of the plasmids for *bla*_CTX−M−3_-positive *K. pneumoniae*.

**34-1.2,15.4Plasmid replicon type^a^**	**Number of isolates (*n*)**	**size approximation (kb) (*n*)**
IncFII(K)	41	216.9 (2), 138.9 (11), 104.5 (21), 78.2 (7)
Col4401	10	138.9 (7), 104.5 (3)
IncQ1	11	104.5 (8), 78.2 (3)
IncFIB(K)	3	173.4 (1), 104.5 (2)
IncFII(pKP91)	1	173.4
IncFII(pKPHS1)	1	138.9
IncR	1	138.9
IncFIIB	1	104.5

a Types of plasmid origins based on the PlasmidFinder analysis of the assembled genome sequences.

### Genetic context of ESBL genes

**Figure 4:**
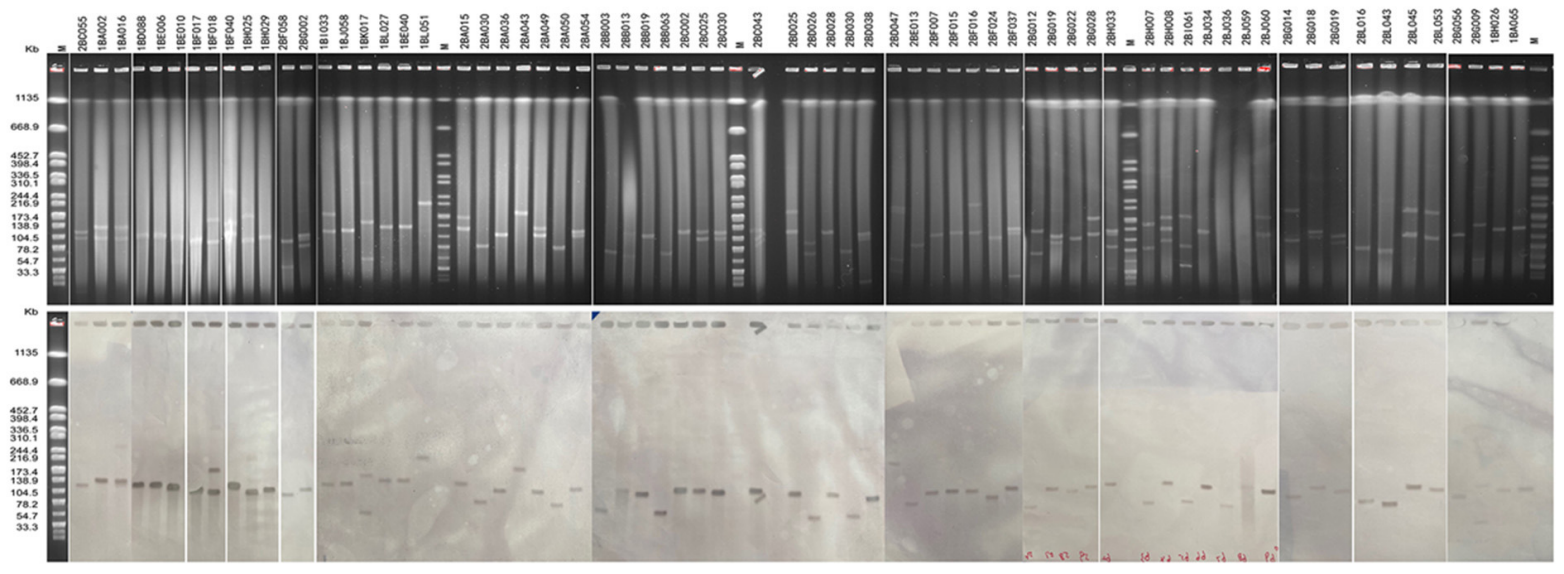
S1-PFGE analysis of *bla*_CTX−M−3_-positive *K. pneumoniae* isolates and southern hybridization. Lane M, *Salmonella* Braenderup H9812 as molecular weight marker.

The *bla*_CTX−M−3_ gene and *bla*_CTX−M−14_ gene surrounding the different isolates showed exactly the same genetic background. The *bla*_CTX−M−14_ gene was flanked by an IS*E062*, IS*1400* and IS*Ecp1* elements upstream and a partial TnAs1 downstream. The presence of the beta-lactam gene (*bla*_TEM_) and tetracycline resistance gene (*tet*) downstream of *bla*_CTX−M−3_. However, genetic variations between *bla*_CTX−M−15_ encoding elements were frequent due to different lengths of the IS*Ecp1* elements, different sizes or locations of the region between IS*Ecp1* and *bla*_CTX−M−15_, and/or a different length of the partial elements (IS*150IV* and Tn*2*). Three distinct gene environments were displayed around the *bla*_CTX−M−55_ gene, whereas ISEcp1 was all located downstream of the gene (Figure 4). Overall, isolates with the same genetic environment appeared in different villages at different times, suggesting the possibility of human-to-human transmission of plasmids carrying this gene.

**Figure 5:**
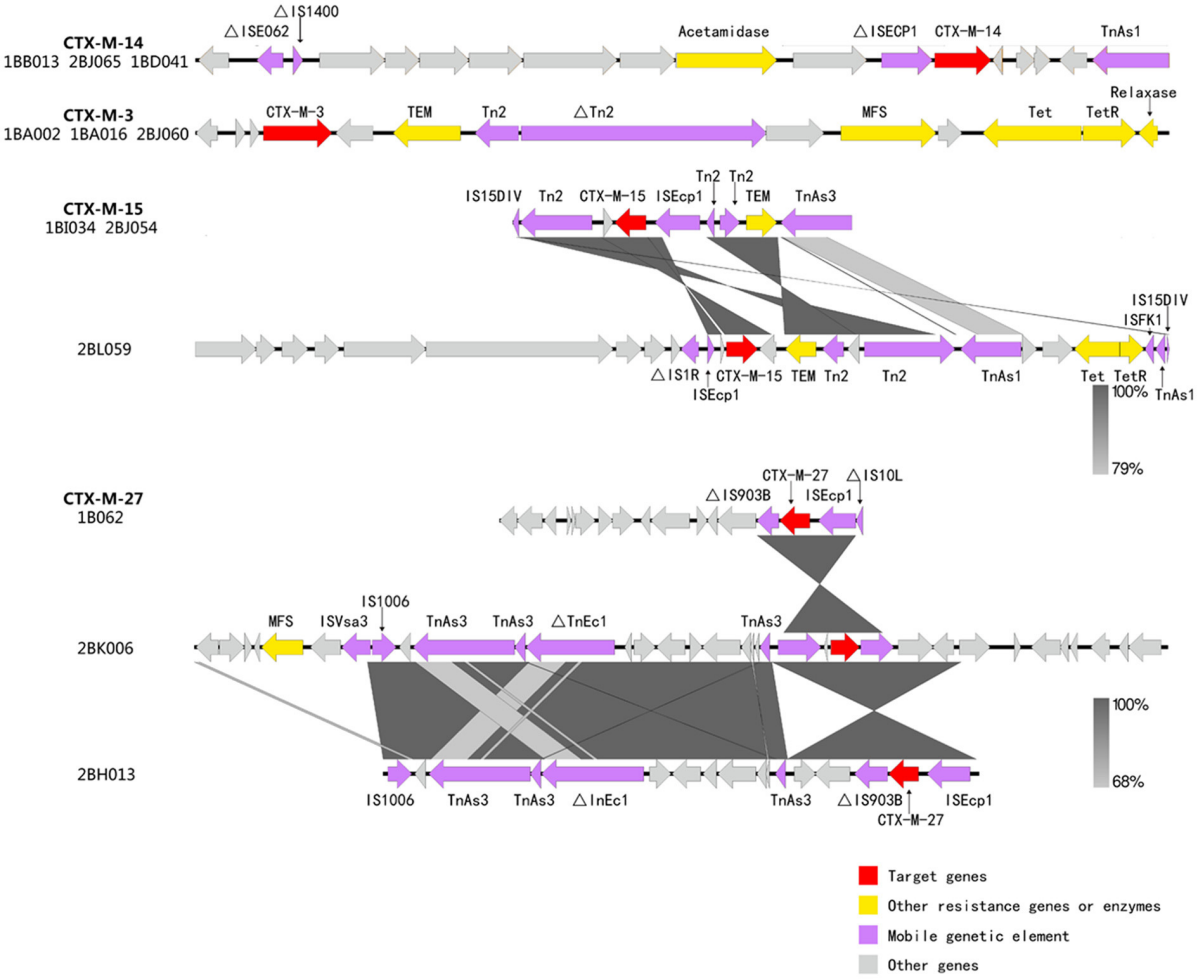
Analysis of the genetic environment surrounding the *bla*_CTX−M_ gene. Arrows represent the direction of transcription. Red filled blocks represent CTX-M, yellow filled blocks represent resistance-related proteins, purple filled blocks represent mobile genetic elements, and gray filled blocks represent other proteins.

### Characterizing ESBL-KP SNPs and phylogeny

**Figure 6:**
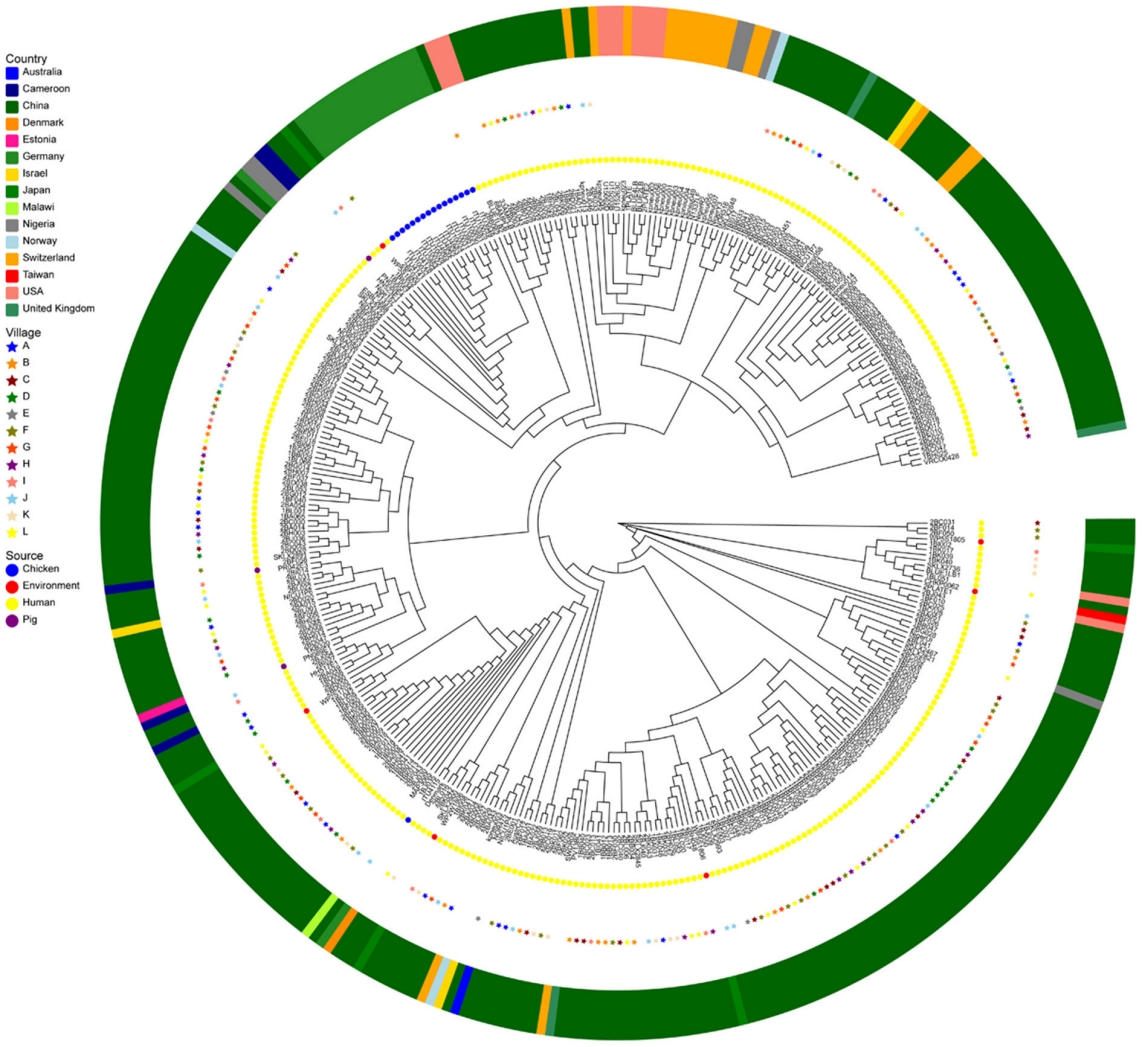
Genomic analysis of ESBLs-producing *K. pneumoniae* (*n* = 264) of this study and *K. pneumoniae* (n = 91) from various regions of the world (*n* = 91). A maximum-likelihood phylogenetic tree was constructed using genome-wide single-nucleotide polymorphism (SNP) analysis. Sources of the isolates are indicated by different colors for location (asterisks or squares) and samples' origin (circles). This core-genome-based maximum-likelihood tree was visualized ignoring information in gene-tree branch lengths for the convenience of discrimination.

The results of the SNP analysis showed that there is a possibility of ESBL-KP transmission in the community. For example, ST659 ESBL-KP isolate 2BC013 recovered in Village C was closely related to 2BC023 retrieved from the same village in 2017 (15 SNPs difference), ST323 ESBL-KP 2BB035 recovered from Village B was closely related to 2BL048 from Village L in 2017 (one SNPs difference), ST776 ESBL-KP 2BF007 and 2BF015 separated in different households from village F in 2017 were closely related (78 SNPs difference). Further, potential ESBL-KP close associations were also detected between individuals in various villages at different times, such as isolates 1BL052 (ST869) from L village and 2BH029 (ST869) from H village (SNPs difference of 5), 1BK025 (ST101) from village K and 2BH055 (ST101) from village H (5 SNPs difference), 1BK025 (ST101) from village K and 2BH055 (ST101) from village H (5 SNPs difference). Surprisingly, no close correlation was found for ESBL-KP from the same villagers. In addition, we compared genome sequences of these isolates to selected ESBL-KP isolated from food, animal, environment and human sources available on the public data base. ST442 ESBL-KP isolates 2BA003 recovered from village A were closely related to the United Kingdom human-derived sample isolate VRC00451 (99 SNPs difference), the remaining 98 isolates were not closely genetically related to the isolates in the study (between 176 and 2098 SNPs) (Figure 6).

## Discussion

Worldwide, the resistance of ESBL-KP to many antibiotics has become a major global problem, which has seriously affected the treatment of infectious diseases (2, 21). In Latin America, ESBLs are produced in approximately 25% of *E. coli* and 53% in *K. pneumoniae*. In Chile, however, the incidence of ESBLs produced by *K. pneumoniae* strains resistant to third-generation cephalosporins is approximately 60% (22, 23). The carrier rate of ESBL-producing *E. coil*/*K. pneumoniae* among veterinarians in the Netherlands is 9.8% (24). In China, the overall positive rate of ESBLs in Enterobacteriaceae from patients with nosocomial urinary tract infection was 37.2% (25). Studies have reported that colonization rates of ESBL-producers are rising in patients and health care worker populations, thereby increasing the size of the repository and increasing the chances of transmission (26, 27). In this study, we used a WGS approach to analyze 264 ESBL-KP isolates collected in two years (2015 and 2017) from the same rural community in China, an Asian country from which data such as these are acutely lacking (19).

**Figure 7:**
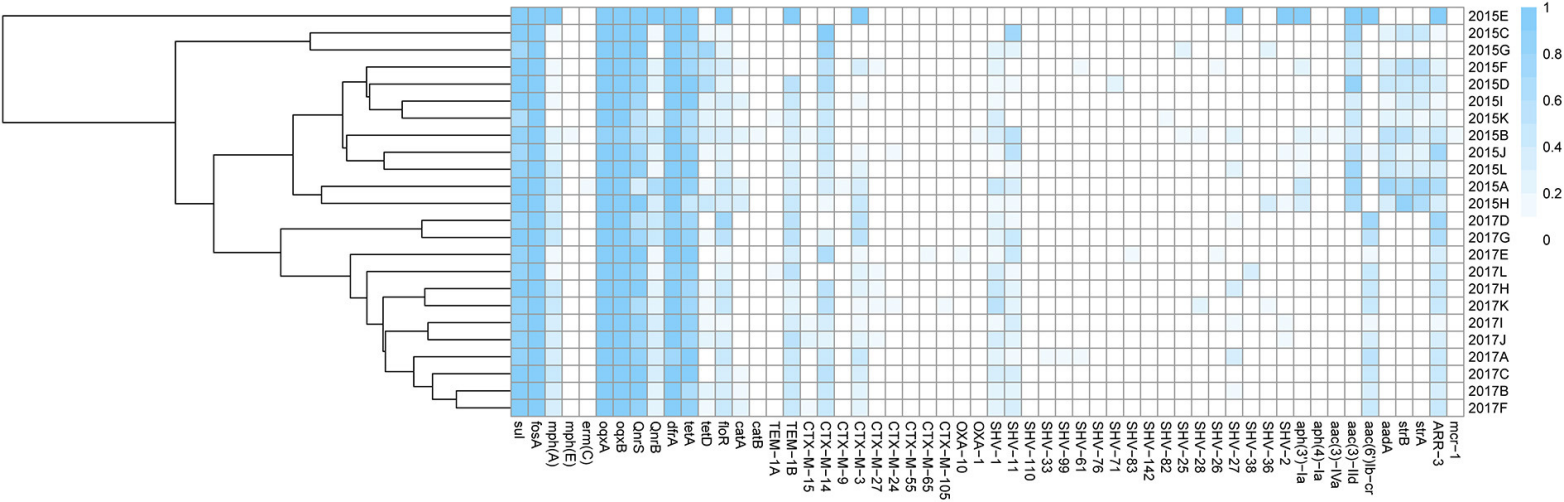
Distribution of phylogenetic group, AMR among ESBL-KP isolates from 12 villages in Shandong Province, China. The color of each box represents the percentage of the resistant gene among sequenced isolates in the corresponding village of the 2 years.

In general, there was a correlation between phenotypic resistance and related genes of ESBL-KP strains isolated in this study. These strains isolated carried a large number of antibiotic resistance genes (Figure 7), including quinolones, aminoglycosides, sulphonamides, trimethoprims, tetracyclines, beta-lactams, phenicols and fluoroquinolones, and it was found that the isolates carrying these resistance genes produced different degrees of resistance to related drugs. All isolates carried quinolone resistance genes, but 26.5% of isolates were not resistant to such drugs, which may be related to the low level of gene expression. Comparing 2015 and 2017, susceptible rates of gentamicin, cefotaxime, ciprofloxacin, ceftazidime, furantoin and trimethoprim-sulfamethoxazole decreased slightly, while susceptible rates of amikacin and tigecycline increased, with the tigecycline increasing rate from 10.90 to 3.80% (Figure 1). Overall, ESBL-KP isolates showed a downward trend in antimicrobial susceptibility. In addition to cefotaxime, susceptible rates were also low (<10%) to trimethoprim-sulfamethoxazole, florfenicol and tetracyclines. Non-susceptibility to these antibiotics corresponded well to carriage of trimethoprim (*dfr*-type), sulphonamide (*sul*-type), phenicol (*floR*-type) and tetracycline resistance genes (tet-type).

All ESBL-KP isolates in this study were not susceptible to florfenicol. Florfenicol is a broad-spectrum antimicrobial exclusively used in veterinary medicine (28). It is still banned for use in humans. The widespread use of florfenicol has led to the emergence and development of cross-resistant pathogens that may enter the human body through the food chain (29). Recently, many florfenicol-resistant bacteria and florfenicol-related resistance genes (*floR*) have been isolated from various animal, human and environmental samples. It is, therefore, speculated that the carriage of this gene, and the lower susceptibility rates, may also be sustained by a selection pressure induced on animals and subsequent run-off into the environment (30, 31). MCR-1 was identified in an ESBL-KP isolate from 2015. The association of *mcr-1* with other resistance mechanisms, such as the production of ESBLs and carbapenemases, which has lead to the development of multidrug-resistant bacteria, could represent a serious clinical problem today (32, 33). The Chinese government has banned the use of polymyxins in animal feed since late 2016 (34). This may be one of the reasons why *mcr-1* was not found in ESBL-KP isolates in 2017 (33).

ST101 has the potential to pose a persistent threat to global public health as an emerging clone that has been identified in many countries and regions. Recent studies have shown that ST101 lineage infection causes an 11% increase in mortality compared with non-ST101 lineage (35). Studies have shown that ST101, a new emerging and extensively resistant sequence type, can further enhance the high risk spreading of carbapenem resistance spreading and polymyxin resistance (36–38). *K. pneumoniae* isolates of type ST101 are closely associated with hospital-acquired infections and epidemics worldwide (39). ST101 and ST274 clones were predominant in ESBL-KP from domestic pets in Italy and France, but ST15 was the most common among ESBL-KP clinical strains from domestic pets in Japan (40). ST3330 clones were dominant in ESBL-KP from an outbreak of sepsis among neonates in an intensive care unit of a China hospital (41). Compared with ESBL-KP obtained in Chinese hospitals and communities, ESBL-KP of healthy people in rural communities showed a different clone distribution. A study from a Chinese hospital showed that ST11 was the most detected ST type in 158 ESBL-KP (42). Another study in a Chinese hospital showed that ST17 is the most popular ST among community-acquired ESBL-KP (2). However, ST101 was the most prevalent one in our study (8.2%). Therefore, the predominant ST in ESBL-KP varies by source sample type and country.

CTX-M-14 and CTX-M-3 were the EABLs with the highest detection rates in our study. This is similar to CTX-M types of ESBL-producing *Raoultella ornithinolytica* and ESBL-KP isolated in environmental samples from the same region (12, 19). This may indicate that bacterial resistance is not only transmitted between the environment and human hosts but also between different bacterial species. The *bla*_CTX−M_ gene has been found on some plasmid types such as InCHI2, IncK, IncF, ZSH6, IncP, IncM2, IncN, IncI and IncL/M (2, 43–45). Our annotated ESBL-KP genome identified incompatibility (Inc) groups of plasmids for IncFIB(pKPHS1), IncFII(K), IncFIA(HI1), IncHI1B, IncHI2A, IncQ1 and IncR. Conjugative plasmids are considered to be one of the important factors for the successful spread of CTX-M ESBLs among various bacteria such as *K. pneumoniae* (46). The hybridization results showed that 93.4% (*n* = 54) of the *bla*_CTX−M−3_ gene was carried by the 78.2–138.9 kb IncF plasmid (Figure 5). The transferability of the plasmids in this study hints at their potential transmission among different host bacteria, and also, due to their wide resistome, this high-risk plasmid may lead to limited treatment options for infected individuals. BLAST analysis showed three isolates (1BB013, 2BJ065 and 1BD041) from different villages at different times showed a similar sequence around *bla*_CTX−M−14_, i.e., ΔIS*E062*-ΔIS*Ecp1*-*bla*_CTX−M−14_-Tn*As1*, which could be related to the lateral transfer of the genetic elements or the clonal expansion of the original host bacteria by some way during this time period. Upstream or downstream of *bla*_CTX−M−15_ and *bla*_CTX−M−27_, insertion of several mobile genetic elements (ISEcp1, IS15DIV and/or IS1006) takes place within Tn2/TnAn3, resulting in truncation of a portion of Tn2/TnAn3 with the blaTEM genes remaining in the loci. The IS insertion may introduce foreign resistance genes into the gene structure, such as tetracycline resistance genes (*tet*) and phenicol resistance genes (*flor*), which we speculate is the result of some antibiotic selection pressure (e.g., florfenicol) (Figure 4).

Our study has several limitations. First, the lack of timely analysis of the isolates in this study led to a long research period. Second, the number of ESBL-KP genomes downloaded from the database was small, so no strains closely related to the genetic relationship of the studied isolates were found.

Altogether, we characterized ESBL-KP strains isolated from the gut of healthy humans in terms of their resistance, resistance genes, and plasmid-mediated transmission mechanisms. Almost all ESBL-KP are multidrug-resistant. All *bla*_CTX−M−3_ were located on the plasmid of ESBL-KP isolates, and the plasmid could transmit between strains from different sources, which presents a new challenge for interrupting the transmission of ESBL-KP in the future. Therefore, it is necessary to further clarify the transmission route of ESBL-KP in this region, strengthen community dialogue, and study the potential health risks associated with it.

## Data availability statement

The datasets presented in this study can be found in online repositories. The names of the repository/repositories and accession number(s) can be found below: https://www.ncbi.nlm.nih.gov/, PRJNA833746.

## Ethics statement

The studies involving human participants were reviewed and approved by Shandong Provincial Center for Disease Control and Prevention Medical Ethics Committee Shandong Provincial Center for Disease Control and Prevention. The patients/participants provided their written informed consent to participate in this study.

## Author contributions

SW: field investigation, data curation, formal analysis, and writing–original draft. ZK: supervision and writing–reviewing and editing. HZ: supervision and funding acquisition. HX: field investigation, validation, and methodology. YC: field investigation, methodology, and software. MF: software and formal analysis. LL: methodology and software. DS: resources and formal analysis. LX, GS, and YL: field investigation. ZB: conceptualization. XY: methodology. BZ: conceptualization, supervision, funding acquisition, and writing-reviewing and editing. All authors contributed to the article and approved the submitted version.
